# Submental Intubation in Panfacial Trauma: A Pictorial Demonstration With Anesthetic Considerations

**DOI:** 10.7759/cureus.106890

**Published:** 2026-04-12

**Authors:** Sri Hari Vignesh R, Leena Sekar, Vivekanandhan Narayanaswamy

**Affiliations:** 1 Department of Anaesthesiology, Palakkad Institute of Medical Sciences, Palakkad, IND; 2 Department of Ophthalmology, Sri Lakshmi Narayana Institute of Medical Sciences, Puducherry, IND

**Keywords:** airway management, difficult airway, mandibular fracture, maxillofacial trauma, nasal fracture, submental intubation

## Abstract

Airway management in patients with panfacial trauma is particularly challenging when both nasal and oral routes are unsuitable. Submental intubation serves as an effective alternative to tracheostomy in such scenarios. We report the case of a 30-year-old male who presented with mandibular and nasal bone fractures following a road traffic accident. Limited mouth opening and the need for intraoperative maxillomandibular fixation made conventional airway approaches unsuitable. Submental intubation was performed after initial oral intubation to facilitate surgical access. The procedure was completed successfully without complications. This case highlights the role of submental intubation as a safe, reliable, and less invasive alternative to tracheostomy in selected maxillofacial trauma cases.

## Introduction

Airway management in patients with maxillofacial trauma presents unique challenges, particularly when both oral and nasal routes are either unsuitable or interfere with surgical access [1]. Nasotracheal intubation may be contraindicated in patients with nasal or midfacial fractures due to the risk of intracranial tube placement and exacerbation of associated injuries [2,3]. Orotracheal intubation, although commonly performed, can interfere with surgical exposure and prevent intraoperative assessment of dental occlusion during mandibular fixation [3]. Tracheostomy has traditionally been used in such situations; however, it is associated with significant early and late complications, including bleeding, infection, pneumothorax, and long-term tracheal stenosis. Therefore, less invasive alternatives are preferred when feasible [3]. Submental intubation, first described by Altemir [1], provides an effective alternative by allowing the endotracheal tube to be exteriorized through the submental region while maintaining airway security and an unobstructed surgical field [4]. It is particularly useful in patients with panfacial trauma where short-term airway control is required without the need for prolonged postoperative ventilation [5,6]. Although submental intubation is a well-established technique and not novel in itself, detailed pictorial demonstration of the procedural steps along with anesthetic considerations remains educationally valuable, particularly for clinicians who may encounter this technique infrequently. We present a case illustrating the practical application of submental intubation in panfacial trauma, with emphasis on technical execution, anesthetic considerations, and perioperative airway management.

## Case presentation

**Figure 1:**
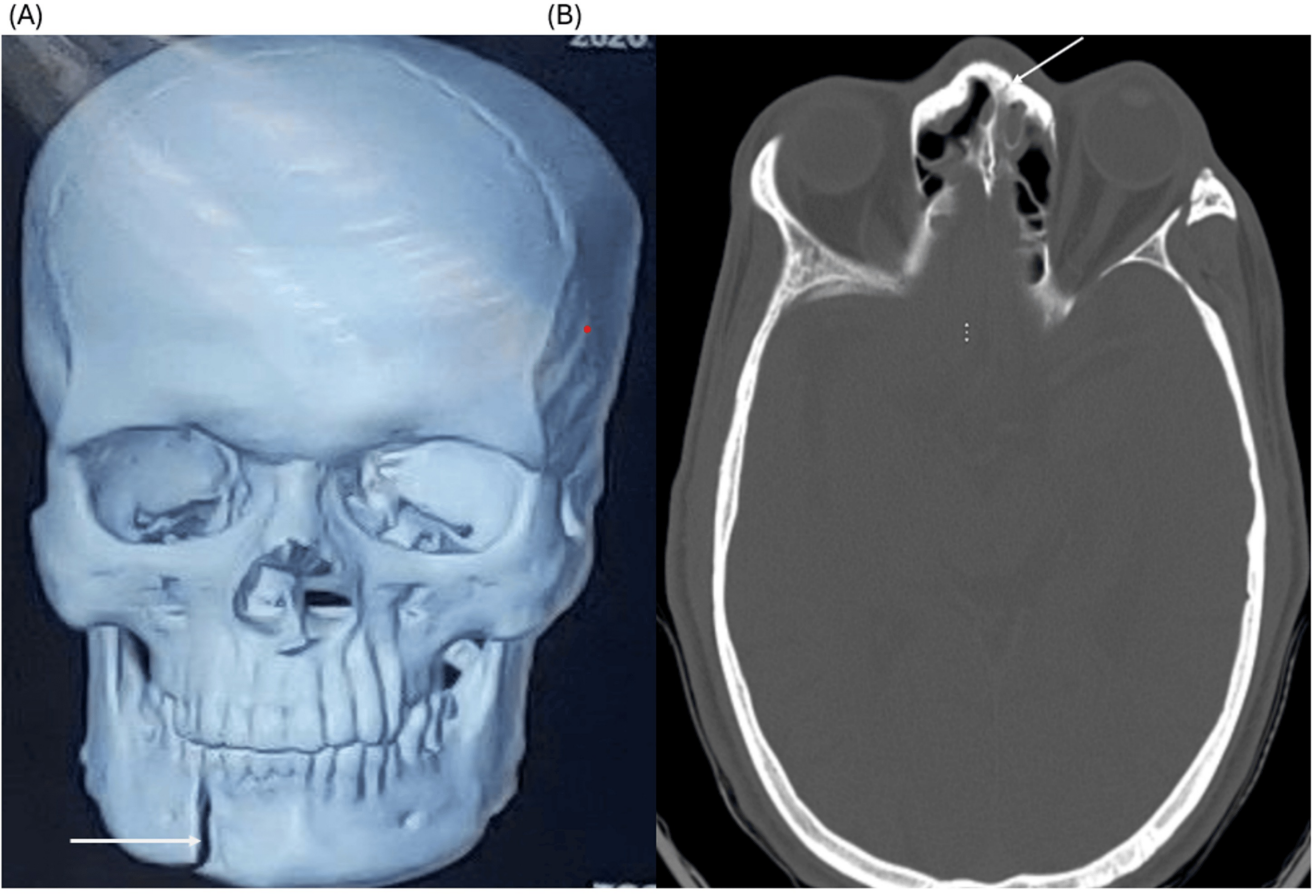
Preoperative CT scan showing facial fractures (A) Three-dimensional reconstruction demonstrating parasymphysis fracture of the mandible (arrow). (B) Axial CT section showing nasal bone fracture (arrow).

A 30-year-old male presented with a history of a fall from a two-wheeler, followed by pain and swelling over the lower jaw and difficulty in mouth opening. There was no history of comorbid illness. On examination, the patient was conscious and oriented with stable vital parameters. Systemic examination revealed normal cardiovascular and respiratory findings, with no focal neurological deficits. Airway assessment suggested a potentially difficult airway, with features including Mallampati grade III and restricted mouth opening of approximately two fingers. Neck movements were adequate. Thyromental distance was adequate, dentition was intact, and no facial asymmetry or cervical spine limitation affecting laryngoscopy was noted. Mask ventilation was anticipated to be feasible. The patient was classified as American Society of Anesthesiologists (ASA) physical status I. Computed tomography of the facial bones revealed a parasymphysis fracture of the mandible along with a nasal bone fracture, making nasotracheal intubation unsuitable (Figure 1).

**Figure 2:**
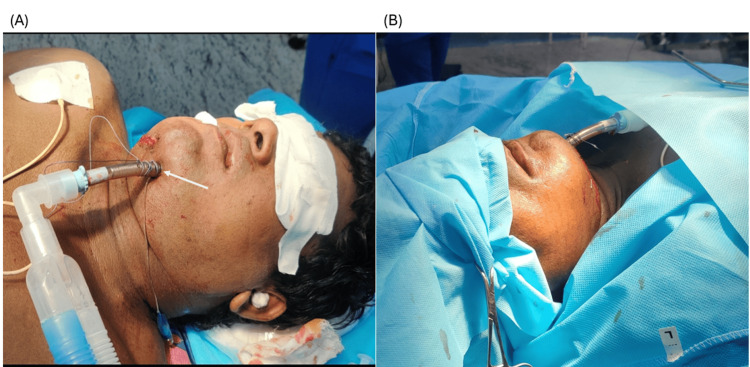
Submental intubation: external view (A) Lateral view showing the endotracheal tube exiting through the submental incision (arrow). (B) Intraoperative view demonstrating the externalized tube in situ, providing an unobstructed surgical field.

After applying standard monitors (ECG, non-invasive blood pressure (NIBP), peripheral oxygen saturation (SpO₂), end-tidal carbon dioxide (ETCO₂), and Temperature), a difficult airway cart was prepared. Premedication was administered with glycopyrrolate 10 mcg/kg IV and fentanyl 2 mcg/kg IV. Anesthesia was induced with thiopentone sodium 5 mg/kg IV and atracurium 0.5 mg/kg IV. Orotracheal intubation was performed by direct laryngoscopy using a Macintosh blade (size 4) with an 8.0 mm flexometallic endotracheal tube, which was secured at 22 cm. Following successful intubation, submental intubation was performed. After throat packing and aseptic preparation of the submental and intraoral regions, local anesthetic infiltration with 2% lignocaine with adrenaline was administered. A 2-cm midline submental incision was made approximately two fingerbreadths below the mandibular border. Blunt dissection was performed through the subcutaneous tissue, platysma, deep cervical fascia, and mylohyoid muscle to create a tract into the floor of the mouth, taking care to remain in the avascular midline plane. A corresponding intraoral mucosal incision was made adjacent to the lingual frenulum. Artery forceps were passed through the tract, and the pilot balloon was exteriorized first, followed by temporary disconnection and exteriorization of the flexometallic tube through the submental tunnel. The breathing circuit was promptly reconnected, and tube position was reconfirmed by capnography and bilateral chest auscultation. The procedure was coordinated with the surgical team to minimize apnea time during tube disconnection and transfer. The tube was secured externally with sutures (Figure 2).

**Figure 3:**
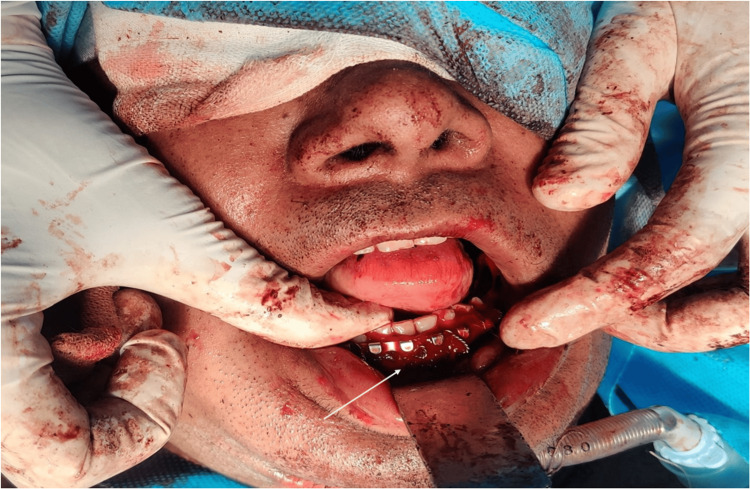
Intraoral view following submental intubation Intraoperative photograph demonstrating exposure of the mandibular fracture site with surgical manipulation of the operative field (arrow).

Anesthesia was maintained with oxygen and nitrous oxide (1:1) along with sevoflurane (1%-2%). Intravenous paracetamol (15 mg/kg) and ketorolac (0.5 mg/kg) were administered for analgesia. The intraoperative period was uneventful. Hemodynamic parameters remained stable throughout the procedure. Estimated blood loss was approximately 300 mL, and the duration of surgery was approximately three hours. Intraoperative findings demonstrated successful fixation of the mandibular fracture with restoration of dental alignment and mandibular contour, while the submental endotracheal tube remained in situ, allowing an unobstructed surgical field (Figure 3).

At the end of the procedure, the endotracheal tube was repositioned back into the oral cavity. The submental incision was closed using 3-0 prolene sutures. Neuromuscular blockade was reversed, and the patient was extubated uneventfully. The postoperative period remained uneventful. The patient was followed until discharge, with satisfactory wound healing and no evidence of infection, salivary fistula, hypertrophic scarring, sensory deficit, or airway-related complications.

## Discussion

**Figure 4:**
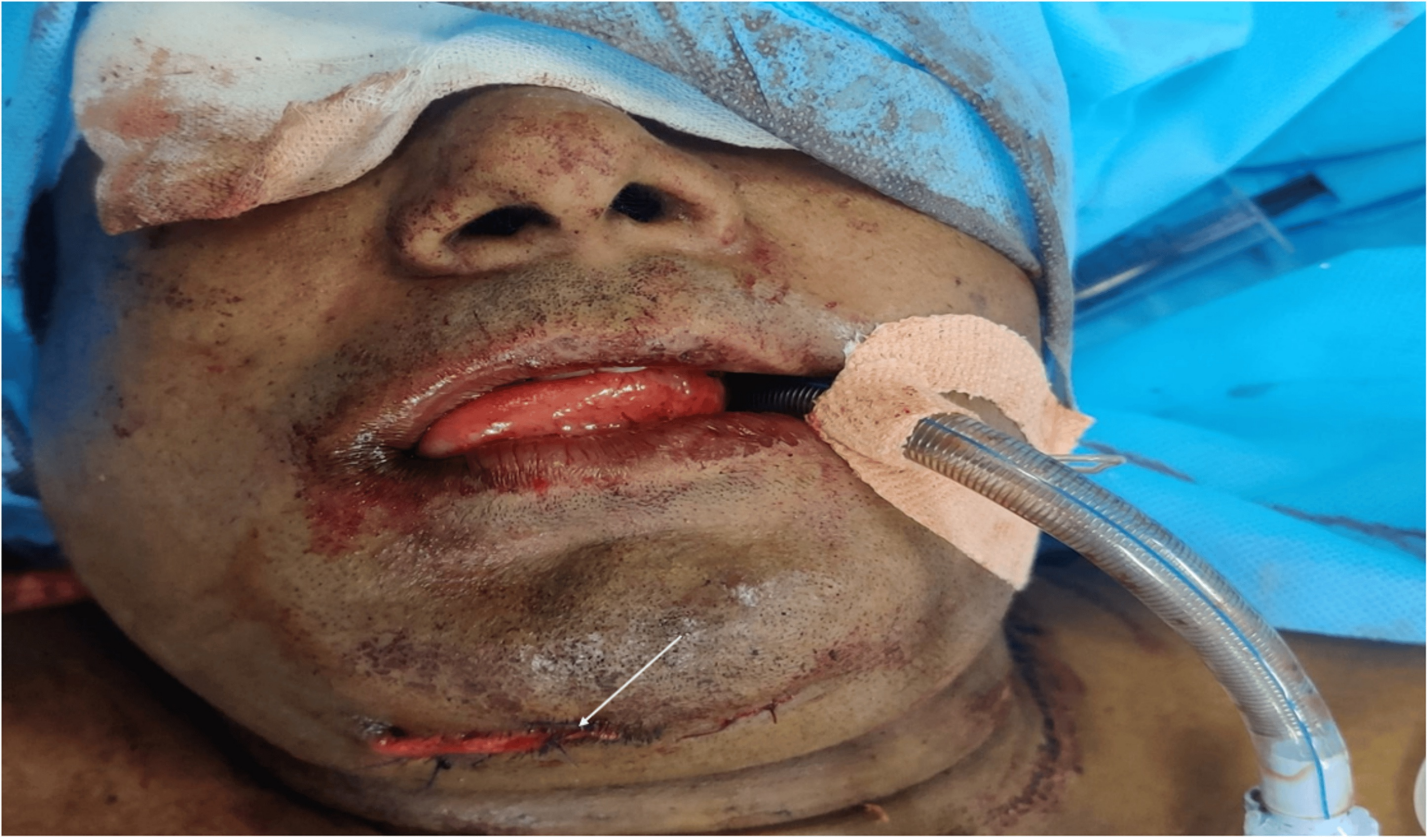
Repositioning of the endotracheal tube into the oral cavity following submental extubation, with closure of the submental incision using sutures (arrow)

Submental intubation is a well-established alternative airway technique in patients with maxillofacial trauma where both oral and nasal routes are unsuitable. In the present case, submental intubation was preferred over tracheostomy because the anticipated duration of postoperative airway support was short, and there was no indication for prolonged mechanical ventilation or repeated airway access. Avoiding tracheostomy reduced the risk of procedure-related morbidity while still permitting adequate surgical exposure. Since its original description by Altemir in 1986, it has gained acceptance as a safe and effective method for airway management in selected cases [1]. In patients with facial fractures, nasotracheal intubation may be contraindicated due to the risk of intracranial placement, particularly in the presence of nasal or skull base injuries [2,3]. Orotracheal intubation, although feasible, may interfere with surgical access and occlusion assessment, which are critical during mandibular fixation [3]. Submental intubation overcomes these limitations by providing a secure airway while allowing unobstructed access to the oral and nasal cavities. Studies have demonstrated that this technique is particularly useful in panfacial trauma and midfacial fractures, where both surgical exposure and airway safety are essential [3,4]. Compared to tracheostomy, submental intubation is less invasive and associated with fewer complications. Tracheostomy carries risks such as hemorrhage, infection, subcutaneous emphysema, pneumothorax, and long-term tracheal stenosis, whereas submental intubation avoids these complications when used for short-term airway management [3,4]. However, the technique is not without risks. Potential complications include damage to the submandibular ducts, lingual nerve, and pilot balloon, as well as infection or scarring at the incision site. Careful midline dissection and meticulous technique are essential to minimize these risks [5,6]. Appropriate patient selection remains critical. Contraindications include the need for prolonged postoperative ventilation, severe neurological injury requiring airway protection, repeated surgeries, or disruption of the floor of mouth anatomy. In this case, direct laryngoscopy provided an adequate view (Cormack-Lehane grade IIa), emphasizing that airway management should be individualized based on clinical assessment and operator expertise. After completion of the procedure, the endotracheal tube was repositioned back into the oral cavity prior to extubation, ensuring controlled airway management and patient safety. Submental intubation is best suited for patients requiring short-duration airway control and is not recommended in cases where prolonged postoperative ventilation is anticipated, in which tracheostomy remains the preferred option [6]. At the end of the procedure, the endotracheal tube was repositioned into the oral cavity. The submental incision was closed using 3-0 prolene sutures, providing a favorable cosmetic outcome with minimal scarring (Figure 4).

## Conclusions

Based on our case experience, submental intubation appears to be a safe and effective alternative to tracheostomy in carefully selected patients with maxillofacial trauma requiring short-term airway control. It provides excellent surgical access while avoiding the morbidity associated with tracheostomy. Successful use of this technique depends on appropriate patient selection, operator expertise, and meticulous procedural execution.
